# Exploration and discovery of treatment targets for primary biliary cholangitis based on plasma and cerebrospinal fluid proteomics: A multicenter mendelian randomization study

**DOI:** 10.1371/journal.pone.0340166

**Published:** 2026-02-12

**Authors:** Jianxin Xi, Shengnan Wang, Jie Chen, Jason Chi Shing Law, Jianglong Wang, Guan Huei Lee, Zhongqi Fan, Guoyue Lv, Yuguo Chen

**Affiliations:** 1 Department of Hepatobiliary and Pancreatic Surgery, General Surgery Center, The First Hospital of Jilin University, ChangChun, Jilin, China; 2 China-Singapore Belt and Road Joint Laboratory on Liver Disease Research, Changchun, China; 3 Department of Neurology, The First Hospital of Jilin University, ChangChun, Jilin, China; 4 Department of Radiology, The First Hospital of Jilin University, Chang Chun, Jilin, China; 5 Department of Neurology, Union Hospital, Tongji Medical College, Huazhong University of Science and Technology, Wuhan, Hubei, China; 6 Faculty of Medicine, The University of Hong Kong, Pok Fu Lam, Hong Kong SAR, China; 7 First Operating Room, The First Hospital of Jilin University, Changchun, Jilin, China; 8 Division of Gastroenterology and Hepatology, Department of Surgery, National University Hospital Singapore, Singapore; PRISM CRO, PAKISTAN

## Abstract

**Background:**

Primary biliary cholangitis (PBC) is a chronic, progressive autoimmune cholestatic liver disease. Current first-line therapy, ursodeoxycholic acid (UDCA), yields suboptimal response rates, leaving many patients at risk of disease progression. Thus, novel therapeutic targets are urgently needed to slow PBC progression.

**Methods:**

We performed two-sample Mendelian randomization (MR) to identify proteins causally associated with PBC risk, using protein quantitative trait loci (pQTL) as genetic instruments. The discovery stage utilized PBC genome-wide association study (GWAS) summary statistics from Cordell et al., followed by replication in independent GWAS datasets from the IEU Open GWAS project and the FinnGen consortium. pQTL data were drawn from large-scale plasma (N = 2,656) and cerebrospinal fluid (CSF; N = 184) proteomic studies. We also conducted sensitivity analyses, including Bayesian colocalization, reverse-causality testing, and phenotype scanning. Identified protein targets were further examined via protein–protein interaction (PPI) network analysis.

**Results:**

Three proteins showed significant associations with PBC risk after Bonferroni correction (*p* < 6.35 × 10^−5^). Elevated plasma *MANBA* (odds ratio [OR] = 1.29, 95% confidence interval [CI] 1.20–1.40, p = 1.22 × 10^−10^) and CSF *TNFSF15* (OR = 6.37, 95% CI 2.94–13.83, p = 2.79 × 10^−6^) were associated with higher PBC risk, whereas elevated plasma *FCRL3* (OR = 0.79, 95% CI 0.73–0.86, p = 2.12 × 10^−8^) was associated with lower risk. Bayesian colocalization analysis indicated that these protein loci and PBC share the same underlying causal variants.

**Conclusion:**

Elevated plasma *MANBA* and CSF *TNFSF15* levels were linked to increased PBC risk, while higher plasma *FCRL3* was protective. Our findings nominate *MANBA* and *TNFSF15* as potential therapeutic targets, while *FCRL3* may serve as a protective biomarker for PBC management.

## Introduction

Primary biliary cholangitis (PBC), previously termed primary biliary cirrhosis until 2016, is a chronic autoimmune liver disorder characterized by progressive cholestasis and inflammatory destruction of intrahepatic bile ducts, ultimately resulting in fibrosis and cirrhosis [1,2]. PBC exhibits substantial geographic variation in its prevalence and incidence, ranging from 3 to 33.8 cases per 100,000 individuals [3–5]. Despite its relatively low prevalence, recent studies have reported a rising trend globally [6]. Immune-mediated injury of bile ducts and chronic cholestasis constitute the primary pathogenic mechanisms of PBC. Diagnosis typically relies on detecting antimitochondrial antibodies (AMA) or PBC-specific antinuclear antibodies (ANA), in conjunction with elevated serum alkaline phosphatase (ALP) levels [7]. AMA are highly specific autoantibodies directed against mitochondrial proteins, while PBC-specific ANA target nuclear components such as sp100 or gp210; both serve as key serological markers for PBC. Although AMA is detected in approximately 90–95% of PBC patients and demonstrates nearly 100% diagnostic specificity, some patients may present without detectable AMA or elevated ALP [2,8,9]. This diagnostic limitation has driven research toward identifying additional biomarkers for improved diagnosis and prognosis prediction [10–14]. While such biomarker studies are crucial, our research shifts the focus distinctly towards therapeutic target discovery, moving beyond diagnostics to address the critical unmet need for novel therapies. Nonetheless, relatively few studies have focused on exploring new therapeutic targets for PBC. Ursodeoxycholic acid (UDCA) is currently the sole first-line therapy for PBC, significantly altering the disease’s natural progression and extending transplant-free survival [15,16]. However, around 40% of patients exhibit inadequate response to UDCA and remain at risk of clinical progression [14]. Second-line treatment with obeticholic acid (OCA) also demonstrates limited effectiveness and poses financial constraints due to high cost [17,18]. Consequently, many patients eventually require liver transplantation (LT). Thus, identifying novel therapeutic targets and effective agents for PBC remains critically important. To comprehensively map potential targets, we performed deep proteomic analysis of plasma and also include the cerebrospinal fluid (CSF) proteome, justified by emerging evidence linking CSF proteins to hepatic autoimmune processes (including gut-brain-liver axis interactions) and their potential to yield unique mechanistic insights into disease pathogenesis.

Proteins present in plasma and cerebrospinal fluid (CSF) are involved in numerous vital biological functions, including signal transduction, transport, and inflammatory responses. Proteomic analyses of these bodily fluids have substantially advanced our understanding of human biological processes [19]. Genome-wide association studies (GWAS) have uncovered thousands of plasma proteins regulated by protein quantitative trait loci (pQTL), facilitating the identification of potential drug targets for various diseases [20–23]. These pQTLs represent genetic variants linked to protein expression levels, with plasma pQTLs specifically reflecting circulating plasma proteins. Therefore, we aim to utilize plasma and CSF pQTL as instrumental variables for large-scale Mendelian randomization (MR) using plasma proteomics to validate the relationship between circulating plasma protein levels and the risk of PBC, to identify new therapeutic targets for PBC [24].

Mendelian randomization (MR) leverages genetic variants as instruments to infer causal relationships between exposures (such as protein levels) and disease outcomes [25–27]. The integration of proteomic data from plasma and CSF with GWAS datasets has expanded the utility of MR in identifying druggable targets for complex diseases [28,29]. However, no previous studies have employed plasma and CSF protein pQTL data within an MR framework to explore therapeutic targets specifically for PBC [30].

**Fig 1:**
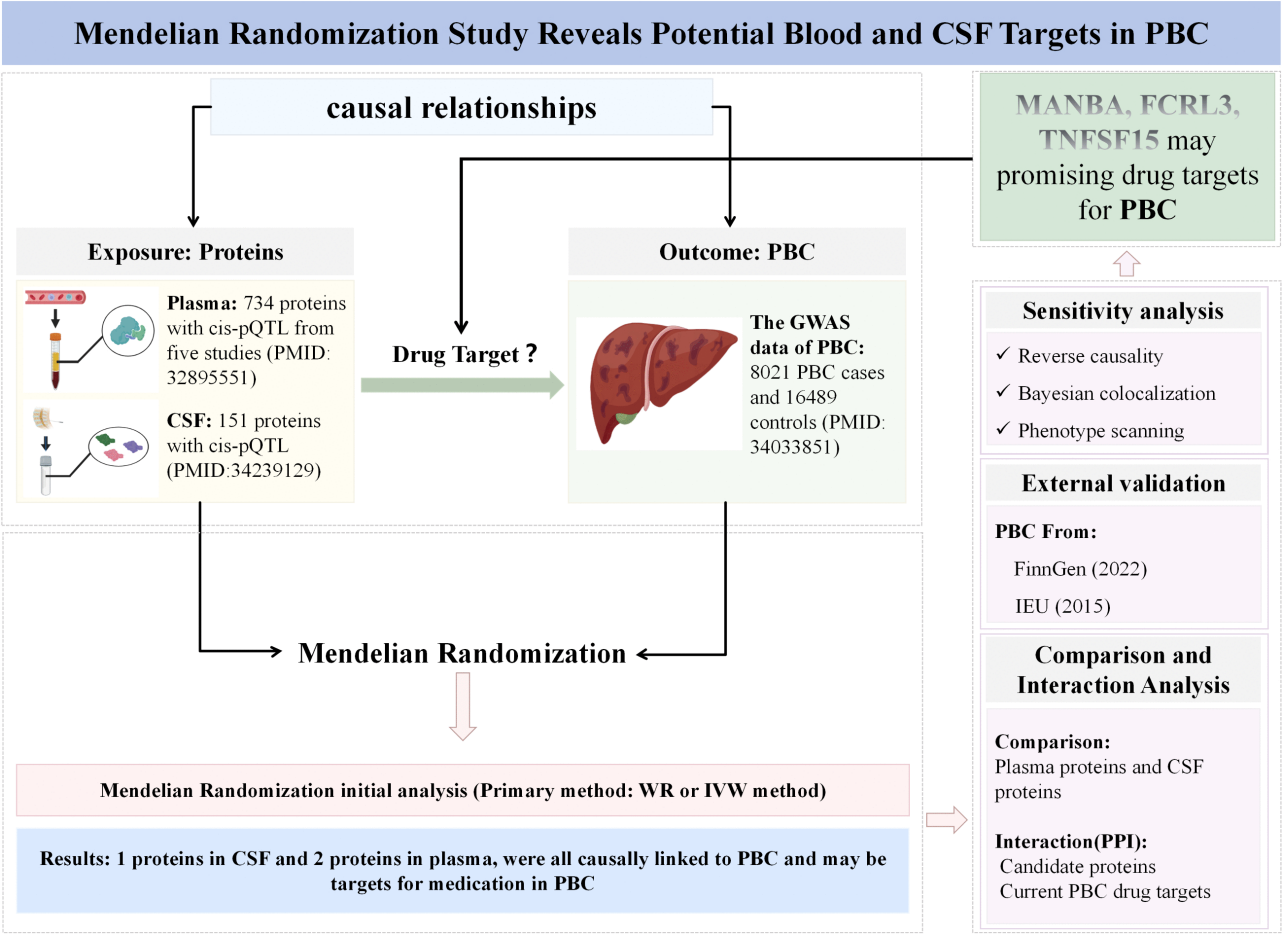
Study design for the identification of plasma and CSF proteins causally associated with PBC.

The aim of this study is to explore potential therapeutic targets for PBC by integrating proteomic data from plasma and CSF within a Mendelian randomization framework. We utilized data from GWAS of PBC individuals from the Cordell study [31], plasma pQTL data from the Zheng study [32], and CSF protein pQTL data from the Yang study [33] to perform MR, aiming to identify causal pathogenic proteins associated with PBC. Subsequently, we conducted rigorous sensitivity analyses and compared the consistency of result directions between the discovery and replication phases. To assess whether these results were influenced by different pathogenic variants in linkage disequilibrium (LD), we also performed co-localization analyses, reverse causality testing, and phenotype scanning. In addition, to explore the interactions between druggable genes prioritized through MR and to further validate their involvement in the pathogenesis of PBC, we conducted PPI analyses on the identified druggable genes. To enhance the credibility of the findings, we performed secondary analyses using GWAS data from the IEU Open GWAS and FinnGen R9 cohorts for external validation. Since the data used in this study were obtained from published literature, it was not necessary to obtain informed consent or ethics approval. The study design is illustrated in Fig 1.

## Materials and methods

### Exposure data acquisition

Since this study utilizes publicly available datasets (including both exposure and outcome data), it does not require ethical approval or informed consent. The primary exposure data consisted of pQTLs for plasma and CSF proteins. Plasma protein data were acquired from a comprehensive proteomic GWAS study by Zheng et al.[32], which meta-analyzed data from five cohort studies comprising predominantly European-ancestry individuals. This study identified 3,606 pQTLs associated with 2,656 plasma proteins [21–23,32].CSF protein data were sourced from Yang et al.[33], who identified 274 pQTLs corresponding to 184 CSF proteins.

For both plasma and CSF proteins, genetic instruments (cis-pQTLs) were selected according to the following criteria:

1. They were cis-acting variants (defined as within ± 1 Mb of the transcription start site of the encoding gene).
2. They achieved genome-wide significance (p < 5 × 10 ⁻ ⁸).
3. Their genomic location was outside the major histocompatibility complex (MHC) region (Chromosome 6: 26–34 Mb).

For closely correlated variants (linkage disequilibrium, LD), the most significant independent SNP was retained after clumping with a stringent LD threshold (r^2^ < 0.001) within a 10,000 kb window to ensure independence and minimize confounding.

Ultimately, 738 cis-pQTLs for 734 plasma proteins (S1 Table) and 154 cis-pQTLs for 151 CSF proteins (S2 Table) were identified for analysis.

### Source of outcome data

The primary outcome data for PBC were obtained from the GWAS meta-analysis by Cordell et al. (2021) [31]. This study comprised 8,021 cases and 16,489 controls of European ancestry, currently representing the largest GWAS dataset available for PBC.

For external validation, additional GWAS datasets from IEU Open GWAS (2,674 PBC cases, 10,475 controls) [34] and FinnGen R9 (ID: finngen_R9_CHIRBIL_PRIM; 557 cases, 281,127 controls) were utilized.

Prior to MR analysis, exposure and outcome datasets were harmonized to ensure effect alleles were aligned. Palindromic SNPs with ambiguous strand orientation (A/T or G/C SNPs) were excluded to avoid allele mismatch. The same genome-wide significance threshold (p < 5 × 10 ⁻ ⁸) was applied consistently for instrument selection across all analyses.

### Mendelian randomization (MR) analysis

#### Main analysis.

MR analyses were performed using plasma and CSF protein levels as exposure variables, with PBC status as the outcome. The primary analysis method employed was the inverse variance weighted (IVW) method, which included multiple instrumental variables for MR studies [35]. For single-instrument analyses, the Wald ratio method was applied [35]. Effect estimates were expressed as odds ratios (ORs), representing the risk of PBC per standard deviation increase in plasma protein levels or per ten-fold increase in CSF protein levels. MR analysis was conducted using the “TwoSampleMR” package in R version 4.2.3. A stringent Bonferroni correction was applied to adjust for multiple testing, establishing a significance threshold at p < 5.65 × 10^−5^ (0.05/885 tests). To enhance the robustness of the genetically supported associations, we conducted a bidirectional MR analysis, utilizing PBC data from the IEU Open GWAS as the exposure. We extracted data related to plasma proteins [22,35] and CSF proteins [33] from three previously published studies as outcome measures. The selection criteria for the exposure factors were consistent with those used in the prior MR analyses. The IVW method was used to check reverse causality and was complemented by MR-Egger regression, Weighted Median (WM), Simple Mode, and Weighted Mode techniques; all these methods were integrated to validate the genetically supported associations [28]. This methodology aids detecting potential reverse causality, minimizing confounding factors, and ensuring the integrity of the genetic association study.

#### Sensitivity analysis.

We performed Steiger filtering to confirm the direction of the associations identified between the proteins and PBC [36]. Steiger filtering posits that an effective instrumental variable should explain more variance in the exposure than in the outcome. If the instrumental variable meets this criterion, its direction is labeled as “TRUE”; otherwise, it is labeled as “FALSE.” After removing any single-nucleotide polymorphisms (SNPs) indicated as having a “FALSE” direction, we repeated all MR analyses using the IVW method.

Bayesian co-localization is a statistical method used to investigate whether the association signals observed in two traits (Trait 1: Potential druggable genes; Trait 2: PBC-related traits) arose from the same genetic variants. This is determined by calculating the posterior probabilities for five hypotheses [37]:

(i) PPH0, where neither trait is associated;
(ii) PPH1, where only Trait 1 is associated with the genetic variant;
(iii) PPH2, where only Trait 2 is associated with the genetic variant;
(iv) PPH3, where both traits are associated with the genetic variant, but these associations arise from different genetic variants; and
(v) PPH4, where both traits are associated with the same genetic variant, and the associations arise from the same genetic variant.

If the posterior probability hypothesis 4 (PPH4) is greater than 80%, it is concluded that the potential druggable gene and PBC share the same genetic variant. We performed Bayesian co-localization analysis using the ‘coloc’ package in R (http://cran.r-project.org/web/packages/coloc).

To control for potential confounding factors and pleiotropic effects associated with the instrumental variables used in this study, we utilized data from extensive genetic association studies and employed the Phenoscanner tool (http://www.phenoscanner.medschl.cam.ac.uk/) to investigate these relationships [38]. To meet the criteria for pleiotropy, it was necessary to initially identify significant genomic associations (*p* < 5 × 10^−8^) for the included instrumental variables. These instrumental factors were derived from GWAS populations of European ancestry. Furthermore, we linked the instrumental variables to risk factors for PBC, such as metabolic traits, protein composition, and clinical characteristics. In addition, we set the r^2^ value for LD of the priority protein pQTLs below 0.001 to minimize the effects of LD. Subsequently, we used the LD link platform to further investigate whether any LD was present [28].

### Analysis of associations between proteins or proteins and drugs

Due to the presence of the blood-brain barrier, we hypothesized that the relationship between the presumed plasma pQTLs and CSF pQTLs may not be strongly correlated. Therefore, we included both plasma and CSF pQTLs in this study and conducted a Spearman correlation analysis to investigate this relationship; the r^2^ value ranges from −1.0 to 1.0; values close to −1.0 indicate a strong negative correlation, while values near 1.0 indicate a strong positive correlation. When the correlation coefficient is close to 0.0, it suggests that there is no significant correlation between the variations in plasma pQTLs and CSF pQTLs. Subsequently, we established different *p*-value thresholds to explore any potential changes in the relationship as the significance level increased. After completing the association analysis between plasma pQTLs and CSF pQTLs, we analyzed the initially identified protein targets potentially associated with PBC risk through PPI networks to assess the interrelated protein targets in plasma and CSF proteins. The PPI network used in this study was obtained from the STRING database (https://cn.string-db.org), a resource for exploring functional protein associations. In the STRING database, the minimum interaction fraction threshold was set at 0.4. Identifying protein target pathways is considered a key approach in discovering effective drug compounds, as drugs not only regulate the activity of specific proteins but also modulate the activity of their downstream effectors to impede disease progression. Therefore, we analyzed the relationships between PBC protein targets, related genes, and approved clinical applications by utilizing the DrugBank database (https://go.drugbank.com/).

## Results

### Multicenter MR analysis revealed that Mannosidase beta (MANBA), Fc receptor-like 3 (FCRL3), and TNF superfamily member 15 (TNFSF15) were significantly associated with the incidence of PBC

**Fig 2:**
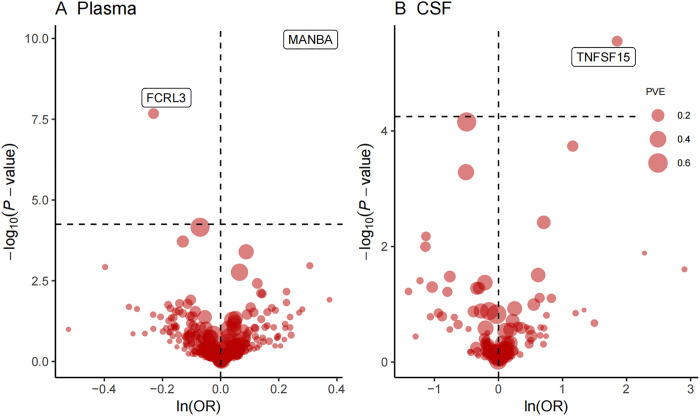
A volcano plot was generated to identify 3 proteins as potential targets in PBC from a pool of 734 plasma proteins (A) and 151 CSF proteins. (B) using the Wald ratio or inverse variance weighted method. CSF: cerebrospinal fluid, PVE: proportion of variance explained.

**Fig 3:**
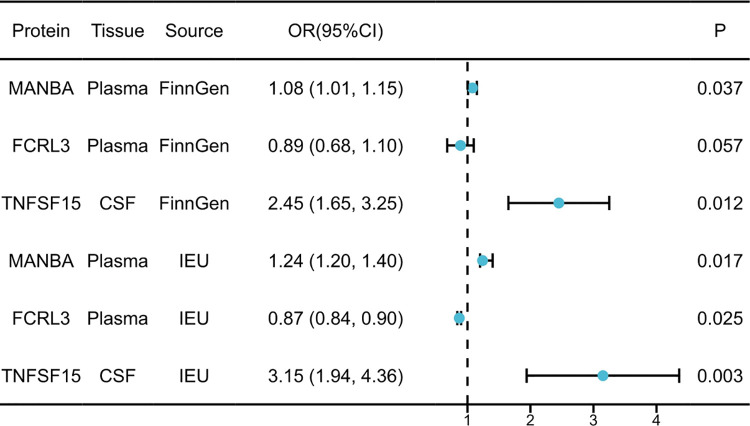
External validation was conducted to assess the genetically supported associations between 3 potential proteins and PBC. Odds ratios (ORs) were calculated to determine the increased risk of PBC per standard deviation (SD) increase in plasma protein levels and per 10-fold increase in CSF protein levels.

A total of 734 plasma protein quantitative trait loci (pQTLs) and 154 cerebrospinal fluid (CSF) pQTLs were analyzed for potential associations with primary biliary cholangitis (PBC). The Bonferroni-corrected threshold was set at *p* < 5.65 × 10^−5^. Three proteins demonstrated significant associations with PBC risk: MANBA and FCRL3 from plasma, and TNFSF15 from CSF (Table 1, Fig 2). Specifically, elevated plasma MANBA levels (odds ratio [OR] = 1.29; 95% confidence interval [CI]: 1.20–1.40; p = 1.22 × 10^-10) and increased CSF TNFSF15 levels (OR = 6.37; 95% CI: 2.94–13.83; p = 2.79 × 10^-6) were significantly associated with a higher risk of PBC. Conversely, elevated plasma FCRL3 levels (OR = 0.79; 95% CI: 0.73–0.86; p = 2.79 × 10^-6) were significantly associated with a reduced risk of PBC. No significant heterogeneity or evidence of horizontal pleiotropy was detected in the primary analyses. To assess the robustness of these findings, external validation was performed using independent GWAS datasets from the IEU Open GWAS and FinnGen consortia. Results from these replication cohorts confirmed consistent associations between MANBA, FCRL3, TNFSF15, and PBC risk (Fig 3).

**Table 1:** MR results for plasma and CSF proteins markedly linked to PBC after Bonferroni-adjusted.

Tissue	Protein	UniProt ID	SNP^a^	Effect allele	OR (95% CI)^b^	P value	PVE	F statistics	Author
Plasma	MANBA	O00462	rs227370	C	1.29 (1.20, 1.40)	1.22e-10	13.36%	509.16	Sun
Plasma	FCRL3	Q96P31	rs7528684	G	0.79 (0.73, 0.86)	2.12e-08	13.34%	508.28	Sun
CSF	TNFSF15	O95150	rs6478109	G	6.37 (2.94, 13.83)	2.79e-06	12.99%	124.61	Yang

^a^All SNPs used were cis-acting.

^b^As the levels of plasma protein increased by one standard deviation, or the levels of CSF protein increased by 10-fold, the risk of PBC also increased.

### Sensitivity analysis confirm a robust and significant link between plasma and CSF proteins, and the risk of PBC

Bidirectional MR, colocalization analysis, and phenotype scanning were performed as sensitivity analyses. MR Steiger filtering initially confirmed robust directional associations for MANBA (p = 4.81 × 10^-76), FCRL3 (p = 3.11 × 10^-78), and TNFSF15 (p = 5.84 × 10^-23) with PBC risk. Bidirectional MR analyses further ruled out reverse causation for these proteins (Table 2).

**Table 2:** Sensitivity analysis on 3 potential causal proteins.

Tissue	Protein	UniProt ID	Steiger filtering	Colocalization PPH4 (coloc.abf)	Phenoscanner
Plasma	MANBA	O00462	TRUE (4.81 × 10^-76^)	0.851	alanine aminotransferase levels
Plasma	FCRL3	Q96P31	TRUE (3.11 × 10^-78^)	0.925	rheumatoid arthritis
CSF	TNFSF15	O95150	TRUE (5.84 × 10^-23^)	0.898	inflammatory bowel disease

Colocalization analyses revealed high posterior probabilities (PPH4 > 0.8) for shared causal variants between PBC risk and MANBA (PPH4 = 0.851), FCRL3 (PPH4 = 0.925), and TNFSF15 (PPH4 = 0.898) (Table 2). MR-Egger regression tests indicated no significant horizontal pleiotropy (all intercepts ~0, p > 0.05; Table 2). Phenotype scanning identified additional associations: plasma MANBA (rs227370) with alanine aminotransferase levels, plasma FCRL3 (rs7528684) with rheumatoid arthritis, and CSF TNFSF15 (rs6478109) with inflammatory bowel disease. Finally, we summarized the above results in Table 2.

**Fig 4:**
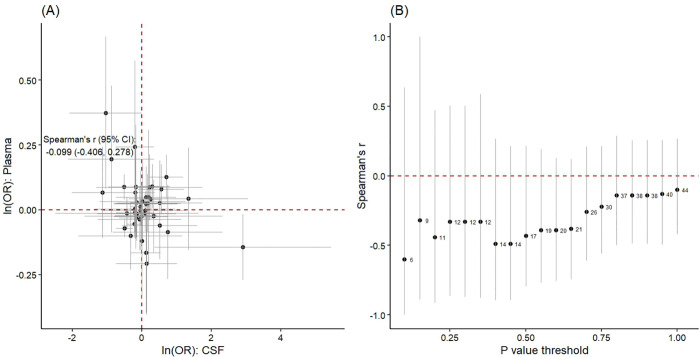
Comparison analysis of MR estimates between plasma proteome and CSF proteome. (A) Correlation analysis was performed on all 92 overlapping proteins in plasma and CSF. The horizontal and vertical gray lines represent 95% confidence intervals for MR estimates. The Spearman correlation coefficient was −0.099 (95% CI: −0.406, 0.278); (B) Spearman’s correlation coefficient was calculated using different cutoffs for P values to include MR estimates. The numbers on the left side of the black point indicated the number of proteins that overlapped.

At the protein level, by comparing and analyzing the pathogenic proteins in plasma and cerebrospinal fluid (CSF) alongside PPI analysis, we obtained a Spearman correlation coefficient of −0.099 (95% CI: −0.406–0.278). This study involved 19 proteins, without a specified *p*-value threshold. Even when different thresholds were applied to limit the number of proteins, significant correlations were still observed (Fig 4). Following the identification of strong associations in the comparative analysis, we proceeded to conduct PPI analysis for the pathogenic proteins identified in both plasma and CSF (S1 Fig and S2 Fig).

### Association between identified therapeutic targets, current treatments, and specific drug candidates for PBC

The STRING database, through experimental validation, revealed robust and reliable interactions between TNFSF15 and FAS, as well as ICOSLG; however, MANBA and FCRL3 were found to exist independently, with no identified interactions with other proteins. Additionally, no interactions were observed among TNFSF15, MANBA, and FCRL3. The STRING database comprises various sources of information, including experimentally validated interactions, computationally predicted interactions, literature mining, and data from other databases. Currently, there is limited research on the role of MANBA and FCRL3 in PBC, which may contribute to the result. Nonetheless, it remains undeniable that investigating the potential therapeutic applications of these compounds for PBC patients within the realm of drug repositioning, as well as studying their mechanisms of action in PBC, represents a valuable pursuit.

## Discussion

In this study, we utilized a two-sample Mendelian randomization (MR) analysis integrating plasma and cerebrospinal fluid (CSF) proteomic data to identify proteins potentially causative for PBC. Our study identified three novel therapeutic targets associated with PBC risk: plasma proteins MANBA and FCRL3, and the CSF protein TNFSF15. Additionally, Bayesian co-localization analyses strongly supported the hypothesis that these proteins and PBC share common underlying genetic variants. We further conducted comprehensive sensitivity analyses, including MR Steiger filtering and phenotype scanning, to verify the robustness of our findings. To validate our findings externally, we performed replication analyses in two additional large-scale GWAS datasets (IEU Open GWAS and FinnGen). These replication results reinforced our initial findings, providing a strong foundation for future therapeutic interventions and targeted preventive strategies for PBC.

MANBA encodes beta-mannosidase, an enzyme implicated in the ultra-rare lysosomal disorder beta-mannosidosis [39]. Beta-mannosidase is localized in the lysosome. Prior cellular and animal studies indicate that MANBA deletion can trigger lysosomal dysfunction, impair autophagic and endocytic pathways, and ultimately cause inflammation-associated cell death and fibrosis [40,41]. Emerging research suggests mitochondrial autophagy may prevent the autoimmune mechanisms that lead to damage of small bile ducts (SBD), a major pathological feature of PBC [42]. Furthermore, in PBC, the bile duct epithelial cells (BECs) in damaged SBD exhibit senescence characteristics and promote increased expression of CCL2 and CX3CL1, which regulate the microenvironment surrounding the bile ducts and participate in the pathogenesis of bile duct injury in PBC [43]. In addition, CCR2-positive cells infiltrate more extensively in the epithelial layer or around the SBD in PBC compared to normal liver tissue, and this infiltration is significantly correlated with CCL2 expression in BEC. This further supports the notion that the chemokine receptor CCL2-CCR2 axis may play a role in the exacerbation of inflammation in PBC [44]. However, a study by Sasaki et al. found that the inhibition of autophagy could significantly downregulate CCL2 and CX3CL1 expression and suppress the senescence of BEC, thus contributing to the pathogenesis of bile duct lesions in PBC [45]. This finding appears to be inconsistent with the earlier conclusions that autophagy might reduce damage to SBD in PBC, potentially due to the differing mechanisms of autophagy. Nonetheless, the finding that MANBA deficiency leads to upregulation of CCL2 remains consistent. In this study, the upregulation of MANBA may increase the risk of developing PBC. It is necessary to further investigate the mechanisms by which MANBA leads to the upregulation of CCL2. Nevertheless, the aforementioned research indicates that MANBA is likely closely associated with the pathogenesis of PBC. Critically, our MR-based discovery approach identifies MANBA not merely as a biomarker associated with PBC, but as a therapeutically actionable target. This contrasts with prior observational studies that primarily established biomarker associations; our findings provide causal inference supporting its direct modulation as a novel therapeutic strategy. Our study extends MANBA’s known role in rare lysosomal storage disorders (beta-mannosidosis) by positioning it as a promising and novel candidate target in the common autoimmune setting of PBC, uniquely revealed by the MR framework.

The Fc receptor-like 3 (FCRL3) gene resides on chromosome 1q21.2-q22 [46]. In an international genomic meta-analysis of PBC conducted by Cordell et al., FCRL3 was similarly identified as a potential risk locus and candidate drug target for PBC [31]. Human FCRL3 is expressed in subpopulations of regulatory T cells (Treg cells), B cells, natural killer cells, CD8 + T cells, and γδ T cells, but it is predominantly expressed in B cells, where it serves as an emerging regulatory factor [46–49]. Accumulating evidence implicates FCRL3 in the maintenance of immune self-tolerance and links it to various autoimmune disorders, such as rheumatoid arthritis, autoimmune thyroid diseases, and systemic lupus erythematosus [50–52]. Currently, research on FCRL3 has primarily focused on its role in T cells and B cells, with little investigation into its functions in other cell types. Treg cells are critical in maintaining immune homeostasis, and dysregulated Treg cell function can lead to the development of autoimmunity [53,54]. In the study by Agarwal et al., it was demonstrated that the activation of FCRL3 can inhibit the immunosuppressive function of Treg cells and promote their transition to a Th17-like phenotype, with sIgA serving as an activating antibody that binds to FCRL3 [55,56]. In PBC, the dominant T cell subsets change as the disease progresses, with Th17 activation becoming significantly predominant in both liver and peripheral blood of PBC patients as well as in animal models during the progressive and late stages of the disease [57–59]. Additionally, the frequency of Treg cells in the peripheral blood and liver of PBC patients is lower than that in healthy controls. By restoring the balance of the Treg/Th17 axis, the progression of disease in PBC models can be halted [60,61]. Importantly, this FCRL3-driven Treg dysfunction and Th17 skewing appears particularly detrimental and central to the hepatic immune dysregulation seen in PBC, potentially reflecting a mechanism more pronounced or uniquely orchestrated within this bile duct-centric autoimmune milieu compared to SLE or RA contexts.

In B cells, FCRL3 possesses both positive and negative signaling motifs and has dual signaling capabilities [62]. Most researchers believe that the expression of FCRL3 on B cells exerts an inhibitory effect on BCR signaling, thereby promoting the breakdown of tolerance in B cells [63,64]. In the liver of PBC patients, there is a significant increase in the proportion of CD19 + B cells [10], leading to the production of higher levels of IL-6, IL-10, IFN-γ, and TNF-α. In addition, the elevated levels of IgM and disease-specific AMA further confirmed that B cell-mediated mechanisms are involved in PBC [65,66]. Critically, the prominent B cell infiltration and localized autoantibody production (notably AMA) within the liver microenvironment represent distinct hallmarks of PBC immunopathology. This provided indirect evidence that FCRL3 may promote the pathogenesis of PBC through B cells. Besides affecting T cells and B cells, SNPs in the FCRL3 promoter region influence NF-κB binding sites, lead to higher protein levels, and an increased susceptibility to a wide range of autoimmune diseases, which may also be related to the pathogenesis of PBC. In summary, targeting Treg cells or B cells may hold potential therapeutic efficacy for PBC [67]. FCRL3 is a surface protein expressed exclusively in lymphocyte subpopulations [55], making it a potential target for therapeutic intervention. Therefore, by modulating FCRL3 signaling, it may be possible to simultaneously regulate the functions of Treg cells and B cells, offering a potential strategy particularly relevant for PBC given its concurrent perturbations in both Treg/Th17 balance and liver-specific B cell responses, mechanisms potentially intertwined or weighted differently compared to other FCRL3-associated diseases like RA or SLE.

CSF may seem unrelated to PBC; however, research has confirmed a possible correlation between HTLV-1-associated myelopathy and transverse myelitis (TM) with the pathogenesis of PBC. The connection between TM and PBC may be overlooked by both neurologists and hepatobiliary disease specialists. Emphasizing the relationship between neuromyelitis optica spectrum disorder and PBC could potentially advance the time window for early immunosuppressive treatment [68,69]. In studies focusing on the correlation between genes and disease, researchers often concentrate solely on the genes alone, neglecting bodily fluids where the transcripts are present. There is a tendency to focus solely on proteins that are commonly found in plasma, while CSF proteins tend to receive attention primarily in the context of neurological disorders, rarely entering the scope of non-neurological diseases. Therefore, in addition to plasma proteomics, this study also incorporates CSF proteomics for comprehensive analysis. Fortunately, we observed a correlation between TNFSF15 in CSF and PBC, suggesting it may serve as a potential therapeutic target for PBC. Notably, the inclusion of the CSF proteome in this study is substantiated by emerging evidence for fluid-crosstalk mechanisms between the central nervous system and the periphery, particularly the liver. Gut-derived inflammatory signals can impact the central nervous system and CSF composition, and emerging research points to bidirectional neuroimmune signaling pathways where CSF constituents, including cytokines like TNFSF15/TL1A, may contribute to hepatic immune modulation and disease pathogenesis in conditions like PBC. TNFSF15, located on chromosome 9q32, encodes the pro-inflammatory cytokine TL1A (TNF superfamily member 15) [70]. In a GWAS, TNFSF15 was identified as the strongest susceptibility gene for PBC beyond the HLA locus, although this finding was initially reported in a Japanese population [71]. Subsequently, this association was also observed in the Han Chinese population [72]. In individuals with the PBC-susceptible allele rs4979462, higher expression levels of endogenous TNFSF15 protein and mRNA can be observed [73]. In similar studies, elevated TNFSF15 levels in PBC patients can provide signals to activate lymphocytes by binding to death receptor 3, or co-stimulate T cells to induce Th1 and Th17 effector cell polarization in response to ligands and immune complexes in monocytes and dendritic cells [74], thereby contributing to the pathogenesis of PBC [75]. These findings align with the results of this study and suggest TNFSF15 may play a crucial role in the pathogenesis of PBC, indicating its potential as a therapeutic target for the treatment of PBC.

The pathogenesis of PBC involves multiple factors, including immune dysfunction, bile acid metabolism, gut microbiota, BEC injury, the gut-liver axis, and fibrosis formation. Several preclinical studies and clinical trials have evaluated therapeutic agents for PBC, but no single agent or mechanism has thus far demonstrated complete effectiveness in halting disease progression and preventing cirrhosis. Therefore, a multi-pronged approach targeting multiple mechanisms and their associated participants is the primary strategy to achieve the ultimate goal of early disease interruption and prolonged LT-free survival [67]. Researchers are also looking to uncover new drug targets from the pathogenic mechanisms of PBC [67,76]. The discovery of new risk loci and the active research of candidate drugs can inject new vitality into various therapeutic mechanisms and approaches for PBC, potentially improving clinical management and outcomes for PBC patients. Proteins represent intermediate phenotypes of the disease, and the associations between their levels and the DNA sequence variations co-localized with common disease risk alleles can expose disease-related pathways, thereby revealing potential drug targets. Proteins or pathway components implicated in this manner may also serve as translational biomarkers [19]. Research findings indicate the potential of MANBA, FCRL3, and TNFSF15 as drug targets for PBC; however, there is currently no relevant research reporting on the agonists or antagonists of these three proteins. Beyond their potential therapeutic role, the altered levels or pathways associated with these proteins may provide valuable insights as biomarkers, aiding in the diagnosis, prognosis, and therapeutic response assessment of PBC patients, as well as in the investigation of disease mechanisms. Furthermore, not limited to PBC itself, we can also explore the therapeutic roles of these three proteins in other diseases, considering the different effects of agonism or antagonism. For example, FCRL3 is believed to be related to the regulatory function of Treg cells. The phenotypic shift of Treg cells from regulatory to inflammatory states can aid in controlling infections and cancer; however, it can also exacerbate autoimmune diseases. Therefore, promoting FCRL3 signaling may benefit cancer immunotherapy and immune responses to pathogens, while inhibiting this signaling could be a strategy for treating autoimmune diseases [55].

In the analysis of this study, we found that MANBA, FCRL3, and TNFSF15 are significantly associated with the risk of PBC. However, our findings remain preliminary, and prior literature has reported variable outcomes for these proteins.

Our study has several limitations that should be considered. For this reason, the proteins identified in this study should be regarded as preliminary candidate targets rather than definitive therapeutic targets, which necessitates more in-depth mechanistic studies for validation. This study assumes valid MR assumptions, but pleiotropy and gene-environment interactions may introduce bias despite sensitivity analyses. Sample selection bias and measurement errors could also affect results. Additionally, it is important to acknowledge that the identified biomarkers may have various sources (either from peripheral or brain), which could influence the results. Therefore, to establish a genetically supported association and to directly inform the choice between agonists or antagonists for these targets, further confirmation through experimental and clinical research will be necessary. Future research can focus on pathway analysis, functional studies using in vitro cell cultures, animal model investigations, and examinations of clinical pathological specimens. These approaches will help to elucidate the biological roles of these proteins in the pathology of PBC and the pathophysiological mechanisms underlying the disease. Ultimately, the development of targeted therapies may involve chemical modifications of candidate drugs to enhance their binding with target proteins, thus improving their efficacy, which will require evaluation and validation through large-scale clinical trials.

## Supporting information

S1 Fig PPI analysis for the pathogenic proteins identified in plasma. (TIF)

S2 Fig PPI analysis for the pathogenic proteins identified in CSF. (TIF)

S1 Table 738 cis-pQTLs involving 734 plasma proteins. (XLSX)

S2 Table 154 cis-pQTLs associated with 151 CSF proteins. (XLSX)

## Abbreviations

PBC: Primary biliary cholangitis
UDCA: ursodeoxycholic acid
MR: Mendelian randomization
CSF: cerebrospinal fluid
PPI: protein-protein interaction
pQTL: protein quantitative trait loci
MANBA: mannosidase, beta D
FCRL3: Fc receptor-like 3
TNFSF15: Tumor Necrosis Factor Superfamily Member 15
GWAS: genome-wide association studies
LD: linkage disequilibrium
IVW: inverse variance weighted
SBD: small bile ducts
BEC: bile duct epithelial cells
AMA: anti-mitochondrial antibodies
SNPs: single nucleotide polymorphisms
BAs: bile acid
LT: liver transplantation
